# Supplementary material for: Polygenetic risk scores do not add predictive power to clinical models for response to anti-TNFα therapy in inflammatory bowel disease
Source: PLoS One. 2021 Sep 17;16(9):e0256860. doi: 10.1371/journal.pone.0256860 (PMC8448323; doi:10.1371/journal.pone.0256860)
Supplement: S1 File — (DOCX) [file pone.0256860.s001.docx]

**Supporting information**

**S1 File. Case and control criteria**

**Primary non-response (PNR)**

Definite PNR: cases required all of the following:

- Non-response after a period up to 16 weeks after starting anti-TNFα therapy accompanied by an alteration of therapeutic approach (addition or escalation of corticosteroids, switch to a different agent, or surgery).
- Non-response was defined by the treating physician, using a combination of clinical, radiologic, endoscopic and laboratory data.
- Received at least standard induction dosing (infliximab 5mg/kg at weeks 0, 2, and 6; adalimumab 160mg at week 0, 80mg at week 2).
- Only response data on their first anti-TNFα exposure was included.

Possible PNR cases required matching of definite case criteria but were adjudicated as a possible PNR case if therapy was continued after 16 weeks of anti-TNFα therapy, despite no clear signs of response. Only response data on their first anti-TNFα exposure was included.

Controls for PNR were all patients with a primary response after 3 infusions with their first anti-TNFα therapy.

**Durable response (DR)**

Definite DR: cases required all of the following:

- Maintenance of response to anti-TNFα therapy for at least 24 months after initiation.
- The response to their first anti-TNFα therapy was defined by a physician’s opinion, using a combination of clinical, radiologic, endoscopic and laboratory data.
- Patients were excluded if available data suggested loss of response after the 24-month time point.
- Only response data on their first anti-TNFα exposure was included.

Probable DR: cases required all of the following:

- Maintenance of response to anti-TNFα therapy for at least 24 months after initiation.
- The response to their first anti-TNFα therapy was defined by a physician’s opinion, using a combination of clinical, radiologic, endoscopic and laboratory data.
- Patients were included if available data suggested loss of response after the 24-month time point.
- Only response data on their first anti-TNFα exposure was included.

Controls for DR were:

- Patients who ceased treatment prior to the 24-month time point due to loss of response.
- Patients who ceased treatment prior to the 24-month time point due to adverse events related to loss of response (such as immunogenicity).
- Patients who ceased treatment prior to the 24-month time point due to adverse events unrelated to loss of response (such as non-IBD related infections) were not included as controls.
- Only response data on their first anti-TNFα exposure was included.
