# Supplementary material for: Polygenetic risk scores do not add predictive power to clinical models for response to anti-TNFα therapy in inflammatory bowel disease
Source: PLoS One. 2021 Sep 17;16(9):e0256860. doi: 10.1371/journal.pone.0256860 (PMC8448323; doi:10.1371/journal.pone.0256860)
Supplement: S2 File — (DOCX) [file pone.0256860.s002.docx]

**Supporting information**

**S2 File. Genetic data generation**

For all patients, DNA was extracted from EDTA-stabilised blood. DNA extraction was performed using the Qiagen Autopure LS (Qiagen NV, Venlo, the Netherlands), and patients were genotyped using the using the Infinium GSA-24 v1.0 BeadChip combined with the optional Multi-Disease drop-in panel (GSA-MD). Genotypes were called using the OptiCall clustering program [1], and quality control steps were performed using PLINK 1.9. First, only autosomal polymorphic variants and variant with a call rate greater than 95% were put forward. Then, individuals with an inbreeding coefficient greater than 0.2, a call rate lower than 98% and sex-mismatches were excluded. Finally, genetic variants with a Hardy-Weinberg equilibrium of P lower than 1 x 10^-6^ were excluded, as were any variants that had a genotyping call rate below 98%. To account for population stratification, principal component analyses were performed using EIGENSTRAT [2] using the 1000 Genomes Project phase I as reference data [3]. Only data from patients clustering with non-Finnish European patients were included in subsequent genetic analyses. Genotype data were phased using the Eagle algorithm and imputed to the Haplotype Reference Consortium reference panel using the Michigan Imputation server [4]. After imputation, 12,130,010 genetic variants with an R^2^ > 0.4 and minor allele frequency greater than 0.1% were used in subsequent analyses.

**References**

Shah TS, Liu JZ, Floyd JA, Morris JA, Wirth N, Barrett JC, Anderson CA. optiCall: a robust genotype-calling algorithm for rare, low-frequency and common variants. Bioinformatics. 2012 Jun 15;28(12):1598-603.

1. Price, A., Patterson, N., Plenge, R. et al. Principal components analysis corrects for stratification in genome-wide association studies. Nat Genet 38, 904–909 (2006).

1000 Genomes Project Consortium, Auton A, Brooks LD, Durbin RM, Garrison EP, Kang HM, Korbel JO, Marchini JL, McCarthy S, McVean GA, Abecasis GR. A global reference for human genetic variation. Nature. 2015 Oct 1;526(7571):68-74.

Das S, Forer L, Schönherr S, et al. Next-generation genotype imputation service and methods. Nat Genet. 2016;48(10):1284-1287.
