# Supplementary material for: Polygenetic risk scores do not add predictive power to clinical models for response to anti-TNFα therapy in inflammatory bowel disease
Source: PLoS One. 2021 Sep 17;16(9):e0256860. doi: 10.1371/journal.pone.0256860 (PMC8448323; doi:10.1371/journal.pone.0256860)
Supplement: S3 File — (DOCX) [file pone.0256860.s003.docx]

**Supporting information**

**S1 File. Case and control criteria**

**Primary non-response (PNR)**

Definite PNR: cases required all of the following:

- Non-response after a period up to 16 weeks after starting anti-TNFα therapy accompanied by an alteration of therapeutic approach (addition or escalation of corticosteroids, switch to a different agent, or surgery).
- Non-response was defined by the treating physician, using a combination of clinical, radiologic, endoscopic and laboratory data.
- Received at least standard induction dosing (infliximab 5mg/kg at weeks 0, 2, and 6; adalimumab 160mg at week 0, 80mg at week 2).
- Only response data on their first anti-TNFα exposure was included.

Possible PNR cases required matching of definite case criteria but were adjudicated as a possible PNR case if therapy was continued after 16 weeks of anti-TNFα therapy, despite no clear signs of response. Only response data on their first anti-TNFα exposure was included.

Controls for PNR were all patients with a primary response after 3 infusions with their first anti-TNFα therapy.

**Durable response (DR)**

Definite DR: cases required all of the following:

- Maintenance of response to anti-TNFα therapy for at least 24 months after initiation.
- The response to their first anti-TNFα therapy was defined by a physician’s opinion, using a combination of clinical, radiologic, endoscopic and laboratory data.
- Patients were excluded if available data suggested loss of response after the 24-month time point.
- Only response data on their first anti-TNFα exposure was included.

Probable DR: cases required all of the following:

- Maintenance of response to anti-TNFα therapy for at least 24 months after initiation.
- The response to their first anti-TNFα therapy was defined by a physician’s opinion, using a combination of clinical, radiologic, endoscopic and laboratory data.
- Patients were included if available data suggested loss of response after the 24-month time point.
- Only response data on their first anti-TNFα exposure was included.

Controls for DR were:

- Patients who ceased treatment prior to the 24-month time point due to loss of response.
- Patients who ceased treatment prior to the 24-month time point due to adverse events related to loss of response (such as immunogenicity).
- Patients who ceased treatment prior to the 24-month time point due to adverse events unrelated to loss of response (such as non-IBD related infections) were not included as controls.
- Only response data on their first anti-TNFα exposure was included.

**S2 File. Genetic data generation**

For all patients, DNA was extracted from EDTA-stabilised blood. DNA extraction was performed using the Qiagen Autopure LS (Qiagen NV, Venlo, the Netherlands), and patients were genotyped using the using the Infinium GSA-24 v1.0 BeadChip combined with the optional Multi-Disease drop-in panel (GSA-MD). Genotypes were called using the OptiCall clustering program [1], and quality control steps were performed using PLINK 1.9. First, only autosomal polymorphic variants and variant with a call rate greater than 95% were put forward. Then, individuals with an inbreeding coefficient greater than 0.2, a call rate lower than 98% and sex-mismatches were excluded. Finally, genetic variants with a Hardy-Weinberg equilibrium of P lower than 1 x 10^-6^ were excluded, as were any variants that had a genotyping call rate below 98%. To account for population stratification, principal component analyses were performed using EIGENSTRAT [2] using the 1000 Genomes Project phase I as reference data [3]. Only data from patients clustering with non-Finnish European patients were included in subsequent genetic analyses. Genotype data were phased using the Eagle algorithm and imputed to the Haplotype Reference Consortium reference panel using the Michigan Imputation server [4]. After imputation, 12,130,010 genetic variants with an R^2^ > 0.4 and minor allele frequency greater than 0.1% were used in subsequent analyses.

**References**

Shah TS, Liu JZ, Floyd JA, Morris JA, Wirth N, Barrett JC, Anderson CA. optiCall: a robust genotype-calling algorithm for rare, low-frequency and common variants. Bioinformatics. 2012 Jun 15;28(12):1598-603.

1. Price, A., Patterson, N., Plenge, R. et al. Principal components analysis corrects for stratification in genome-wide association studies. Nat Genet 38, 904–909 (2006).

1000 Genomes Project Consortium, Auton A, Brooks LD, Durbin RM, Garrison EP, Kang HM, Korbel JO, Marchini JL, McCarthy S, McVean GA, Abecasis GR. A global reference for human genetic variation. Nature. 2015 Oct 1;526(7571):68-74.

Das S, Forer L, Schönherr S, et al. Next-generation genotype imputation service and methods. Nat Genet. 2016;48(10):1284-1287.

**S1 Table.**

**Table 1. Single-nucleotide polymorphisms associated with primary non-response in patients with Crohn’s disease.**

| Chromosome | SNP | Risk  allele | Freq. PNR^a^ | Freq.  PR^a^ | P - value^b^ | Odds ratio^b^ |
| --- | --- | --- | --- | --- | --- | --- |
| 1 | rs3766606 | T | 0.300 | 0.167 | 0.0217 | 0.317 |
| 1 | rs4845604 | A | 0.133 | 0.188 | 0.0019 | 2.463 |
| 2 | rs6708413 | G | 0.167 | 0.287 | 0.0447 | 1.712 |
| 3 | rs3197999 | A | 0.300 | 0.287 | 0.0248 | 0.505 |
| 3 | rs9847710 | C | 0.567 | 0.380 | 0.0131 | 0.506 |
| 3 | rs17200795 | G | 0.100 | 0.149 | 4.60E-05 | 3.193 |
| 3 | rs2045307 | C | 0.233 | 0.213 | 7.40E-05 | 2.785 |
| 6 | rs2503322 | A | 0.267 | 0.430 | 0.0412 | 0.591 |
| 7 | rs1182188 | C | 0.167 | 0.281 | 0.0144 | 1.859 |
| 8 | rs921720 | A | 0.333 | 0.369 | 0.0499 | 0.586 |
| 9 | rs4246905 | T | 0.367 | 0.201 | 0.0470 | 1.660 |
| 10 | rs10761659 | A | 0.500 | 0.425 | 0.0407 | 1.659 |
| 12 | rs7956809 | G | 0.133 | 0.127 | 4.30E-05 | 3.204 |
| 16 | rs1728785 | A | 0.233 | 0.238 | 0.0445 | 0.498 |
| 18 | rs8083571 | A | 0.500 | 0.450 | 2.40E-05 | 2.948 |

SNPs were selected in a prior study at p-value < 0.05 among 163 IBD risk alleles and p-value of <1 × 10^-4^ among the immunochip. For the weighted analysis of PRS we used the previously calculated odds ratios [1].

a = our study in CD

b = the prior study in CD

Abbreviations: SNP, single-nucleotide polymorphism; Freq., Frequency; PNR, primary non-response; PR, primary response; IBD, inflammatory bowel disease; CD, Crohn’s disease.

**S2 Table.**

**Table 2. Single-nucleotide polymorphisms associated with durable response in patients with Crohn’s disease.**

| Chromosome | SNP | Risk  allele | Freq. DR^a^ | Freq. LOR^a^ | P - value^b^ | Odds ratio^b^ |
| --- | --- | --- | --- | --- | --- | --- |
| 1 | rs2651244 | A | 0.400 | 0.314 | 0.0409 | 1.509 |
| 2 | rs1440088 | G | 0.178 | 0.157 | 0.0355 | 1.737 |
| 2 | rs12994997 | G | 0.378 | 0.386 | 0.0476 | 0.682 |
| 5 | rs254560 | A | 0.465 | 0.414 | 0.0271 | 0.651 |
| 6 | rs17119 | G | 0.130 | 0.114 | 0.0472 | 1.701 |
| 6 | rs212388 | C | 0.457 | 0.471 | 0.0137 | 0.622 |
| 7 | rs9297145 | C | 0.252 | 0.257 | 0.0456 | 1.572 |
| 9 | rs55689715 | C | 0.230 | 0.214 | 6.00E-05 | 3.324 |
| 10 | rs12722515 | A | 0.178 | 0.186 | 0.0020 | 2.997 |
| 11 | rs11229555 | T | 0.265 | 0.143 | 0.0100 | 0.579 |
| 12 | rs2682714 | C | 0.330 | 0.386 | 6.10E-05 | 2.438 |
| 14 | rs194749 | C | 0.248 | 0.243 | 0.0491 | 1.663 |
| 16 | rs35725751 | T | 0.165 | 0.171 | 9.30E-05 | 0.451 |
| 16 | rs7201929 | C | 0.170 | 0.171 | 8.10E-05 | 0.451 |
| 17 | rs9904253 | A | 0.361 | 0.357 | 9.80E-05 | 0.466 |
| 20 | rs6087990 | C | 0.400 | 0.471 | 0.0133 | 1.637 |

SNPs were selected in a prior study at p-value < 0.05 among 163 IBD risk alleles and p-value of <1 × 10^-4^ among the immunochip. For the weighted analysis of PRS we used the previously calculated odds ratios [1].

a = our study in CD

b = the prior study in CD

Abbreviations: SNP, single-nucleotide polymorphism; Freq., Frequency; DR, durable response; LOR, loss of response; IBD, inflammatory bowel disease; CD, Crohn’s disease.

**S3 Table.**

**Table 3. Single-nucleotide polymorphisms associated with primary non-response in patients with ulcerative colitis.**

| Chromosome | SNP | Risk  allele | Freq. PNR^a^ | Freq.  PR^a^ | P - value^b^ | Odds ratio^b^ |
| --- | --- | --- | --- | --- | --- | --- |
| 1 | rs6679677 | A | 0.083 | 0.074 | 0.041 | 2.26 |
| 6 | rs3851228 | T | 0.042 | 0.088 | 0.027 | 2.23 |
| 9 | rs4743820 | C | 0.333 | 0.287 | 0.044 | 1.81 |
| 11 | rs568617 | T | 0.208 | 0.176 | 0.042 | 0.39 |
| 12 | rs653178 | C | 0.583 | 0.493 | 0.049 | 1.78 |
| 13 | rs3742130 | A | 0.125 | 0.243 | 0.023 | 1.98 |
| 21 | rs2284553 | A | 0.458 | 0.382 | 0.037 | 1.80 |
| 9 | rs1330307 | C | 0.542 | 0.471 | 5.65E-06 | 0.23 |

SNPs were selected in a prior study at p-value < 0.05 among 201 IBD risk alleles and p-value of <1 × 10^-6^ among the immunochip. For the weighted analysis of PRS we used the previously calculated odds ratios [2].

a = our study in UC

b = the prior study in UC

Abbreviations: SNP, single-nucleotide polymorphism; Freq. Frequency; PNR, primary non-response; PR, primary response; IBD, inflammatory bowel disease; UC, ulcerative colitis.

**S4 Table.**

**Table 4. Single-nucleotide polymorphisms associated with durable response in patients with ulcerative colitis.**

| Chromosome | SNP | Risk  allele | Freq DR^a^ | Freq LOR^a^ | P - value^b^ | Odds ratio^b^ |
| --- | --- | --- | --- | --- | --- | --- |
| 1 | rs670523 | A | 0.316 | 0.275 | 0.021 | 0.64 |
| 2 | rs6716753 | C | 0.211 | 0.200 | 0.026 | 0.61 |
| 4 | rs4692386 | T | 0.421 | 0.450 | 0.004 | 0.57 |
| 7 | rs1077773 | G | 0.316 | 0.400 | 0.035 | 0.68 |
| 8 | rs921720 | A | 0.421 | 0.275 | 0.042 | 0.67 |
| 10 | rs2790216 | A | 0.132 | 0.175 | 0.048 | 1.56 |
| 11 | rs907611 | A | 0.237 | 0.275 | 0.039 | 1.54 |
| 16 | rs529866 | T | 0.211 | 0.125 | 0.001 | 2.18 |
| 16 | rs5743289 | T | 0.132 | 0.275 | 0.033 | 1.79 |
| 17 | rs3091315 | G | 0.316 | 0.300 | 0.024 | 0.63 |
| 18 | rs9319943 | C | 0.316 | 0.100 | 0.037 | 1.66 |
| 16 | rs12051532 | C | 0.395 | 0.325 | 8.44E-06 | 2.35 |

SNPs were selected in a prior study at p-value < 0.05 among 201 IBD risk alleles and p-value of <1 × 10^-6^ among the immunochip. For the weighted analysis of PRS we used the previously calculated odds ratios [2].

a = our study in UC

b = the prior study in UC

Abbreviations: SNP, single-nucleotide polymorphism; Freq. Frequency; DR, durable response; LOR, loss of response; IBD, inflammatory bowel disease; UC, ulcerative colitis.

**References**

1. Barber GE, Yajnik V, Khalili H, Giallourakis C, Garber J, Xavier R, et al. Genetic Markers Predict Primary Non-Response and Durable Response To Anti-TNF Biologic Therapies in Crohn's Disease. Am J Gastroenterol. 2016 Dec;111(12):1816-1822. doi: 10.1038/ajg.2016.408. Epub 2016 Sep 6. PMID: 27596696; PMCID: PMC5143156.
2. Burke KE, Khalili H, Garber JJ, Haritunians T, McGovern DPB, Xavier RJ, et al. Genetic Markers Predict Primary Nonresponse and Durable Response to Anti-Tumor Necrosis Factor Therapy in Ulcerative Colitis. Inflamm Bowel Dis. 2018 Jul 12;24(8):1840-1848. doi: 10.1093/ibd/izy083. PMID: 29718226; PMCID: PMC6128143.
