# Supplementary material for: Polygenetic risk scores do not add predictive power to clinical models for response to anti-TNFα therapy in inflammatory bowel disease
Source: PLoS One. 2021 Sep 17;16(9):e0256860. doi: 10.1371/journal.pone.0256860 (PMC8448323; doi:10.1371/journal.pone.0256860)
Supplement: S1 Table — SNPs were selected in a prior study at p-value < 0.05 among 163 IBD risk alleles and p-value of <1 × 10^–4^ among the immunochip. For the weighted analysis of PRS we used the previously calculated odds ratios [1]. a = our study in CD. b = the prior study in CD. Abbreviations: SNP, single-nucleotide polymorphism; Freq., Frequency; PNR, primary non-response; PR, primary response; IBD, inflammatory bowel disease; CD, Crohn’s disease. (DOCX) [file pone.0256860.s004.docx]

**Supporting information**

**S1 Table.**

**Table 1. Single-nucleotide polymorphisms associated with primary non-response in patients with Crohn’s disease.**

| Chromosome | SNP | Risk  allele | Freq. PNR^a^ | Freq.  PR^a^ | P - value^b^ | Odds ratio^b^ |
| --- | --- | --- | --- | --- | --- | --- |
| 1 | rs3766606 | T | 0.300 | 0.167 | 0.0217 | 0.317 |
| 1 | rs4845604 | A | 0.133 | 0.188 | 0.0019 | 2.463 |
| 2 | rs6708413 | G | 0.167 | 0.287 | 0.0447 | 1.712 |
| 3 | rs3197999 | A | 0.300 | 0.287 | 0.0248 | 0.505 |
| 3 | rs9847710 | C | 0.567 | 0.380 | 0.0131 | 0.506 |
| 3 | rs17200795 | G | 0.100 | 0.149 | 4.60E-05 | 3.193 |
| 3 | rs2045307 | C | 0.233 | 0.213 | 7.40E-05 | 2.785 |
| 6 | rs2503322 | A | 0.267 | 0.430 | 0.0412 | 0.591 |
| 7 | rs1182188 | C | 0.167 | 0.281 | 0.0144 | 1.859 |
| 8 | rs921720 | A | 0.333 | 0.369 | 0.0499 | 0.586 |
| 9 | rs4246905 | T | 0.367 | 0.201 | 0.0470 | 1.660 |
| 10 | rs10761659 | A | 0.500 | 0.425 | 0.0407 | 1.659 |
| 12 | rs7956809 | G | 0.133 | 0.127 | 4.30E-05 | 3.204 |
| 16 | rs1728785 | A | 0.233 | 0.238 | 0.0445 | 0.498 |
| 18 | rs8083571 | A | 0.500 | 0.450 | 2.40E-05 | 2.948 |

SNPs were selected in a prior study at p-value < 0.05 among 163 IBD risk alleles and p-value of <1 × 10^-4^ among the immunochip. For the weighted analysis of PRS we used the previously calculated odds ratios [1].

a = our study in CD

b = the prior study in CD

Abbreviations: SNP, single-nucleotide polymorphism; Freq., Frequency; PNR, primary non-response; PR, primary response; IBD, inflammatory bowel disease; CD, Crohn’s disease.

**References**

1. Barber GE, Yajnik V, Khalili H, Giallourakis C, Garber J, Xavier R, et al. Genetic Markers Predict Primary Non-Response and Durable Response To Anti-TNF Biologic Therapies in Crohn's Disease. Am J Gastroenterol. 2016 Dec;111(12):1816-1822. doi: 10.1038/ajg.2016.408. Epub 2016 Sep 6. PMID: 27596696; PMCID: PMC5143156.
2. Burke KE, Khalili H, Garber JJ, Haritunians T, McGovern DPB, Xavier RJ, et al. Genetic Markers Predict Primary Nonresponse and Durable Response to Anti-Tumor Necrosis Factor Therapy in Ulcerative Colitis. Inflamm Bowel Dis. 2018 Jul 12;24(8):1840-1848. doi: 10.1093/ibd/izy083. PMID: 29718226; PMCID: PMC6128143.
