# Supplementary material for: Polygenetic risk scores do not add predictive power to clinical models for response to anti-TNFα therapy in inflammatory bowel disease
Source: PLoS One. 2021 Sep 17;16(9):e0256860. doi: 10.1371/journal.pone.0256860 (PMC8448323; doi:10.1371/journal.pone.0256860)
Supplement: S2 Table — SNPs were selected in a prior study at p-value < 0.05 among 163 IBD risk alleles and p-value of <1 × 10^–4^ among the immunochip. For the weighted analysis of PRS we used the previously calculated odds ratios [1]. a = our study in CD. b = the prior study in CD. Abbreviations: SNP, single-nucleotide polymorphism; Freq., Frequency; DR, durable response; LOR, loss of response; IBD, inflammatory bowel disease; CD, Crohn’s disease. (DOCX) [file pone.0256860.s005.docx]

**Supporting information**

**S2 Table.**

**Table 2. Single-nucleotide polymorphisms associated with durable response in patients with Crohn’s disease.**

| Chromosome | SNP | Risk  allele | Freq. DR^a^ | Freq. LOR^a^ | P - value^b^ | Odds ratio^b^ |
| --- | --- | --- | --- | --- | --- | --- |
| 1 | rs2651244 | A | 0.400 | 0.314 | 0.0409 | 1.509 |
| 2 | rs1440088 | G | 0.178 | 0.157 | 0.0355 | 1.737 |
| 2 | rs12994997 | G | 0.378 | 0.386 | 0.0476 | 0.682 |
| 5 | rs254560 | A | 0.465 | 0.414 | 0.0271 | 0.651 |
| 6 | rs17119 | G | 0.130 | 0.114 | 0.0472 | 1.701 |
| 6 | rs212388 | C | 0.457 | 0.471 | 0.0137 | 0.622 |
| 7 | rs9297145 | C | 0.252 | 0.257 | 0.0456 | 1.572 |
| 9 | rs55689715 | C | 0.230 | 0.214 | 6.00E-05 | 3.324 |
| 10 | rs12722515 | A | 0.178 | 0.186 | 0.0020 | 2.997 |
| 11 | rs11229555 | T | 0.265 | 0.143 | 0.0100 | 0.579 |
| 12 | rs2682714 | C | 0.330 | 0.386 | 6.10E-05 | 2.438 |
| 14 | rs194749 | C | 0.248 | 0.243 | 0.0491 | 1.663 |
| 16 | rs35725751 | T | 0.165 | 0.171 | 9.30E-05 | 0.451 |
| 16 | rs7201929 | C | 0.170 | 0.171 | 8.10E-05 | 0.451 |
| 17 | rs9904253 | A | 0.361 | 0.357 | 9.80E-05 | 0.466 |
| 20 | rs6087990 | C | 0.400 | 0.471 | 0.0133 | 1.637 |

SNPs were selected in a prior study at p-value < 0.05 among 163 IBD risk alleles and p-value of <1 × 10^-4^ among the immunochip. For the weighted analysis of PRS we used the previously calculated odds ratios [1].

a = our study in CD

b = the prior study in CD

Abbreviations: SNP, single-nucleotide polymorphism; Freq., Frequency; DR, durable response; LOR, loss of response; IBD, inflammatory bowel disease; CD, Crohn’s disease.

**References**

1. Barber GE, Yajnik V, Khalili H, Giallourakis C, Garber J, Xavier R, et al. Genetic Markers Predict Primary Non-Response and Durable Response To Anti-TNF Biologic Therapies in Crohn's Disease. Am J Gastroenterol. 2016 Dec;111(12):1816-1822. doi: 10.1038/ajg.2016.408. Epub 2016 Sep 6. PMID: 27596696; PMCID: PMC5143156.
2. Burke KE, Khalili H, Garber JJ, Haritunians T, McGovern DPB, Xavier RJ, et al. Genetic Markers Predict Primary Nonresponse and Durable Response to Anti-Tumor Necrosis Factor Therapy in Ulcerative Colitis. Inflamm Bowel Dis. 2018 Jul 12;24(8):1840-1848. doi: 10.1093/ibd/izy083. PMID: 29718226; PMCID: PMC6128143.
