# Supplementary material for: Polygenetic risk scores do not add predictive power to clinical models for response to anti-TNFα therapy in inflammatory bowel disease
Source: PLoS One. 2021 Sep 17;16(9):e0256860. doi: 10.1371/journal.pone.0256860 (PMC8448323; doi:10.1371/journal.pone.0256860)
Supplement: S3 Table — SNPs were selected in a prior study at p-value < 0.05 among 201 IBD risk alleles and p-value of <1 × 10^–6^ among the immunochip. For the weighted analysis of PRS we used the previously calculated odds ratios [2]. a = our study in UC. b = the prior study in UC. Abbreviations: SNP, single-nucleotide polymorphism; Freq. Frequency; PNR, primary non-response; PR, primary response; IBD, inflammatory bowel disease; UC, ulcerative colitis. (DOCX) [file pone.0256860.s006.docx]

**Supporting information**

**S3 Table.**

**Table 3. Single-nucleotide polymorphisms associated with primary non-response in patients with ulcerative colitis.**

| Chromosome | SNP | Risk  allele | Freq. PNR^a^ | Freq.  PR^a^ | P - value^b^ | Odds ratio^b^ |
| --- | --- | --- | --- | --- | --- | --- |
| 1 | rs6679677 | A | 0.083 | 0.074 | 0.041 | 2.26 |
| 6 | rs3851228 | T | 0.042 | 0.088 | 0.027 | 2.23 |
| 9 | rs4743820 | C | 0.333 | 0.287 | 0.044 | 1.81 |
| 11 | rs568617 | T | 0.208 | 0.176 | 0.042 | 0.39 |
| 12 | rs653178 | C | 0.583 | 0.493 | 0.049 | 1.78 |
| 13 | rs3742130 | A | 0.125 | 0.243 | 0.023 | 1.98 |
| 21 | rs2284553 | A | 0.458 | 0.382 | 0.037 | 1.80 |
| 9 | rs1330307 | C | 0.542 | 0.471 | 5.65E-06 | 0.23 |

SNPs were selected in a prior study at p-value < 0.05 among 201 IBD risk alleles and p-value of <1 × 10^-6^ among the immunochip. For the weighted analysis of PRS we used the previously calculated odds ratios [2].

a = our study in UC

b = the prior study in UC

Abbreviations: SNP, single-nucleotide polymorphism; Freq. Frequency; PNR, primary non-response; PR, primary response; IBD, inflammatory bowel disease; UC, ulcerative colitis.

**References**

1. Barber GE, Yajnik V, Khalili H, Giallourakis C, Garber J, Xavier R, et al. Genetic Markers Predict Primary Non-Response and Durable Response To Anti-TNF Biologic Therapies in Crohn's Disease. Am J Gastroenterol. 2016 Dec;111(12):1816-1822. doi: 10.1038/ajg.2016.408. Epub 2016 Sep 6. PMID: 27596696; PMCID: PMC5143156.
2. Burke KE, Khalili H, Garber JJ, Haritunians T, McGovern DPB, Xavier RJ, et al. Genetic Markers Predict Primary Nonresponse and Durable Response to Anti-Tumor Necrosis Factor Therapy in Ulcerative Colitis. Inflamm Bowel Dis. 2018 Jul 12;24(8):1840-1848. doi: 10.1093/ibd/izy083. PMID: 29718226; PMCID: PMC6128143.
