# Supplementary material for: Polygenetic risk scores do not add predictive power to clinical models for response to anti-TNFα therapy in inflammatory bowel disease
Source: PLoS One. 2021 Sep 17;16(9):e0256860. doi: 10.1371/journal.pone.0256860 (PMC8448323; doi:10.1371/journal.pone.0256860)
Supplement: S4 Table — SNPs were selected in a prior study at p-value < 0.05 among 201 IBD risk alleles and p-value of <1 × 10^–6^ among the immunochip. For the weighted analysis of PRS we used the previously calculated odds ratios [2]. a = our study in UC. b = the prior study in UC. Abbreviations: SNP, single-nucleotide polymorphism; Freq. Frequency; DR, durable response; LOR, loss of response; IBD, inflammatory bowel disease; UC, ulcerative colitis. (DOCX) [file pone.0256860.s007.docx]

**Supporting information**

**S4 Table.**

**Table 4. Single-nucleotide polymorphisms associated with durable response in patients with ulcerative colitis.**

| Chromosome | SNP | Risk  allele | Freq DR^a^ | Freq LOR^a^ | P - value^b^ | Odds ratio^b^ |
| --- | --- | --- | --- | --- | --- | --- |
| 1 | rs670523 | A | 0.316 | 0.275 | 0.021 | 0.64 |
| 2 | rs6716753 | C | 0.211 | 0.200 | 0.026 | 0.61 |
| 4 | rs4692386 | T | 0.421 | 0.450 | 0.004 | 0.57 |
| 7 | rs1077773 | G | 0.316 | 0.400 | 0.035 | 0.68 |
| 8 | rs921720 | A | 0.421 | 0.275 | 0.042 | 0.67 |
| 10 | rs2790216 | A | 0.132 | 0.175 | 0.048 | 1.56 |
| 11 | rs907611 | A | 0.237 | 0.275 | 0.039 | 1.54 |
| 16 | rs529866 | T | 0.211 | 0.125 | 0.001 | 2.18 |
| 16 | rs5743289 | T | 0.132 | 0.275 | 0.033 | 1.79 |
| 17 | rs3091315 | G | 0.316 | 0.300 | 0.024 | 0.63 |
| 18 | rs9319943 | C | 0.316 | 0.100 | 0.037 | 1.66 |
| 16 | rs12051532 | C | 0.395 | 0.325 | 8.44E-06 | 2.35 |

SNPs were selected in a prior study at p-value < 0.05 among 201 IBD risk alleles and p-value of <1 × 10^-6^ among the immunochip. For the weighted analysis of PRS we used the previously calculated odds ratios [2].

a = our study in UC

b = the prior study in UC

Abbreviations: SNP, single-nucleotide polymorphism; Freq. Frequency; DR, durable response; LOR, loss of response; IBD, inflammatory bowel disease; UC, ulcerative colitis.

**References**

1. Barber GE, Yajnik V, Khalili H, Giallourakis C, Garber J, Xavier R, et al. Genetic Markers Predict Primary Non-Response and Durable Response To Anti-TNF Biologic Therapies in Crohn's Disease. Am J Gastroenterol. 2016 Dec;111(12):1816-1822. doi: 10.1038/ajg.2016.408. Epub 2016 Sep 6. PMID: 27596696; PMCID: PMC5143156.
2. Burke KE, Khalili H, Garber JJ, Haritunians T, McGovern DPB, Xavier RJ, et al. Genetic Markers Predict Primary Nonresponse and Durable Response to Anti-Tumor Necrosis Factor Therapy in Ulcerative Colitis. Inflamm Bowel Dis. 2018 Jul 12;24(8):1840-1848. doi: 10.1093/ibd/izy083. PMID: 29718226; PMCID: PMC6128143.
